# Ethics, economics and the regulation and adoption of new medical devices: case studies in pelvic floor surgery

**DOI:** 10.1186/1472-6939-11-14

**Published:** 2010-08-26

**Authors:** Sue Ross, Charles Weijer, Amiram Gafni, Ariel Ducey, Carmen Thompson, Rene Lafreniere

**Affiliations:** 1 Department of Obstetrics and Gynaecology, University of Calgary, Calgary, Canada; 2 Department of Surgery, University of Calgary, Calgary, Canada; 3 Departments of Community Health Sciences and Family Medicine, University of Calgary, Calgary, Canada; 4 Departments of Philosophy and Medicine, Joseph L. Rotman Institute of Science and Values, University of Western Ontario, London, Canada; 5 Department of Clinical Epidemiology and Biostatistics and the Centre for Health Economics and Policy Analysis, McMaster University, Hamilton, Canada; 6 Department of Sociology, University of Calgary, Calgary, Canada

## Abstract

**Background:**

Concern has been growing in the academic literature and popular media about the licensing, introduction and adoption of surgical devices before full effectiveness and safety evidence is available to inform clinical practice. Our research will seek empirical survey evidence about the roles, responsibilities, and information and policy needs of the key stakeholders in the introduction into clinical practice of new surgical devices for pelvic floor surgery, in terms of the underlying ethical principals involved in the economic decision-making process, using the example of pelvic floor procedures.

**Methods/Design:**

Our study involves three linked case studies using, as examples, selected pelvic floor surgery devices representing Health Canada device safety risk classes: low, medium and high risk. Data collection will focus on stakeholder roles and responsibilities, information and policy needs, and perceptions of those of other key stakeholders, in seeking and using evidence about new surgical devices when licensing and adopting them into practice. For each class of device, interviews will be used to seek the opinions of stakeholders. The following stakeholders and ethical and economic principles provide the theoretical framework for the study:

**Stakeholders **- federal regulatory body, device manufacturers, clinicians, patients, health care institutions, provincial health departments, and professional societies. Clinical settings in two centres (in different provinces) will be included.

**Ethics **- beneficence, non-maleficence, autonomy, justice.

**Economics **- scarcity of resources, choices, opportunity costs.

For each class of device, responses will be analysed to compare and contrast between stakeholders. Applied ethics and economic theory, analysis and critical interpretation will be used to further illuminate the case study material.

**Discussion:**

The significance of our research in this new area of ethics will lie in providing recommendations for regulatory bodies, device manufacturers, clinicians, health care institutions, policy makers and professional societies, to ensure surgical patients receive sufficient information before providing consent for pelvic floor surgery. In addition, we shall provide a wealth of information for future study in other areas of surgery and clinical management, and provide suggestions for changes to health policy.

## Background

A US Food and Drug Administration (FDA) public health announcement, released to physicians in October 2008, highlighted concerns associated with the use of licensed mesh devices in the minimally invasive surgical repair of pelvic organ prolapse and stress urinary incontinence. The announcement reported serious complications resulting from the use of licensed devices from nine manufacturers, and pointed out that some of the complications led to significant decrease in patient quality of life with ongoing symptoms. The FDA memorandum stressed the need for surgeons to seek specialized training for each type of technique, to be vigilant about adverse events and complications, and highlighted the importance of informing patients about the potential for adverse events with possible long-term unpleasant effects on quality of life [1]. The information in the FDA announcement was reiterated in 2010 by the Health Canada Marketed Health Products Directorate [2]. These unusual announcements underline the dangers associated with the adoption of new licensed medical devices, and provide a reminder that the responsibility for patient outcome lies not only with regulators and device manufacturers, but also with policy makers and surgeons, and that patient should be active in the decision to have surgery performed.

The decisions surrounding the choice of medical device to approve (by device regulators), fund (by health care funding bodies) or adopt into clinical practice (by hospital authorities and clinicians) are affected by many different motivators and barriers, such as perceived effectiveness, safety (as highlighted by the FDA and Health Canada [1,2]), and also ethical and economic considerations. The research described in this protocol will examine specifically how ethics and economics influence stakeholders' decisions about adopting new devices, particularly when full information on effectiveness and safety may not be available.

In Canada, the licensing and use of new medical devices occurs within a health care system in which the adoption of new technologies is guided by ethics and economics as well as by clinical investigation: assuming that there is evidence that a new device is effective, the adoption of that device is subject to ethical and economic choices at all levels of the health care system. Ethics influences such decisions, seeking assurance that the device will benefit patients while not doing them disproportionate harm, that individuals are fully informed before they make decisions about adopting a new device, and that risks and benefits of a new treatment (or from withdrawing access to an older one) are fairly and reasonably distributed [3]. Economic theory offers further insights into the decision-making process, suggesting that in situations of limited resources, choices are necessary about which technologies should be adopted and which services should be sacrificed in order to free up resources [3-6]. Thus both ethics and economics are concerned with making good choices in adopting new health technologies, and may even be considered inseparable [3-6]. They are as fundamental to decisions about licensing and adopting new clinical devices, as considerations of clinical effectiveness and safety.

### Licensing medical devices in Canada

The introduction of new medical devices in Canada is regulated by the *Medical Devices Regulations *of the *Food and Drugs Act *[7]. The process is governed by the Medical Device Regulatory Framework and undertaken by the Medical Device Bureau (MDB, one of the bureaux of the Health Canada Therapeutic Products Division). The mandate of MDB is specifically to evaluate and monitor the safety, efficacy and quality of diagnostic and therapeutic medical devices [8]. Manufacturers of medical devices require licenses to sell their products in Canada [9]: each new surgical device is classified according to risk, based on factors such as degree of invasiveness, duration of contact with patient, energy transmission hazard and consequences of device malfunction or failure. Risk class (Table 1) is allocated by comparing new devices to similar or competitive devices [9].

**Table 1:** Medical Device Bureau classification of medical devices

MDB Class	Risk	Examples
**Class I**	Lowest risk	Surgical instruments, culture media
**Class II**	Low risk	Contact lenses, epidural catheters, pregnancy test kits, surgical gloves, ultrasound scanner
**Class III**	Moderate risk	Orthopaedic implants, glucose monitors, dental implants, haemodialysis systems
**Class IV**	High risk	HIV test kits, pacemakers, angiographic catheters

The level and amount of evidence required for licensing a specific new device (or group of devices) depends on the classification of risk assigned to that device: the greatest degree of rigour is required for evidence of safety and efficacy in Class IV devices. Class I devices do not remain in contact with the patient for long periods, so the risks are deemed low. Manufacturers of Class I devices are not required to obtain licenses for specific devices, but need only an "establishment license" [9]. For new Class II to Class IV devices, licenses may be approved without evidence of safety or effectiveness, if the new device is "substantially equivalent" to a licensed device, or is a "family member" of an already licensed product [7,10]. The licensing regulations in the USA and European Union are similar in allowing approval without clinical testing [10,11].

### Ethical issues associated with introducing new medical devices

We recently discussed the ethical issues associated with the introduction of new surgical devices, using new urogynaecological devices as an example [12]. Our key concern was that newer devices are being marketed at an earlier stage in the development process, before adequate research evidence of effectiveness and safety is available to inform clinical practice.

Our study was novel in analysing the ethical issues from six perspectives: MDB, device manufacturers, clinicians, patients, health care institutions and professional societies. We found that, in the case of new "family members" (i.e. new surgical devices made by a manufacturer who already produces licensed devices for the same clinical indication), rigorous evidence of safety is not required for MDB to license the new device, even though the use of such a device may be potentially harmful to patients. The lack of clinical evidence impacted on the ethical behaviour of all parties, calling for particular care in making open disclosures of the absence of information and experience. Our evaluation concluded that, without more rigorous MDB requirements for safety and effectiveness information for licensing devices, manufacturers and clinicians should aim for higher ethical standards to protect the health and safety of patients requiring surgical interventions. If not required by MDB, manufacturers should cooperate with physicians in designing and undertaking appropriate clinical research, and should provide balanced presentations of all the available evidence (or lack if it). Before using a new surgical device, clinicians should carefully evaluate the available evidence of effectiveness and safety and seek training in the use of the device. They should provide patients with a careful evaluation of the evidence and inform patients about their personal experience with the new device. Patients should be encouraged to decide themselves, based on full information about the new device and their surgeon's experience, whether they wish to receive the new device. We also suggested that MDB should require rigorous clinical trial evidence before licensing new family members, similar to the level of evidence required for licensing new types of device [12].

### Economic issues

Our preliminary evaluation raised a number of unanswered questions. In particular, we did not consider the economic perspective that would focus on exploring whether the adoption of a new procedure, for which effectiveness and safety have not been adequately established, would represent efficient use of limited societal resources. In addition, we did not consider the opportunity costs to Health Canada and the device manufacturers of increasing the level of evidence required for licensing new devices. Further our evaluation was not sufficiently detailed to define the important ethical and economic questions in regulating medical devices.

Much published literature describes the issues surrounding ethics, economics and regulation and adoption of drugs [13-19]. Little comparable work has examined these issues in relation to the regulation and adoption of medical devices, although several authors have pointed out the paucity of health economics and ethics support for health technology assessments of medical devices [20-22].

The disciplines of ethics and economics offer complementary insights into the decision-making context, and our proposed study will therefore undertake a detailed examination of the regulation and introduction of new medical devices into clinical practice, integrating ethical and economic principles to guide the work.

### Research Question

What are the roles, responsibilities, and information and policy needs of the key stakeholders in the introduction into clinical practice of new surgical devices, in terms of the underlying ethical principles involved in the economic decision-making process, using the example of pelvic floor procedures?

## Methods/Design

The proposed study will consist of three linked case studies using, as examples, selected pelvic floor surgery devices representing Health Canada device safety risk classes: low risk (eg suture capturing device), moderate risk (eg mesh device for pelvic organ prolapse repair) and high risk (eg implantable sacral nerve stimulator). Data collection will focus on stakeholder roles and responsibilities, information and policy needs, and perceptions of those of other key stakeholders, in seeking and using evidence about new surgical devices when licensing and adopting them into practice. For each class of device, interviews will be used to survey the judgements and experiences of stakeholders. The study will be undertaken as an investigation of the conduct and beliefs of the stakeholders from their perspective, but will also use applied ethics and economics theories, arguments and analyses to illuminate the case studies. Ethical approval for the study was received from the University of Calgary Conjoint Health Research Ethics Board (E-22708).

### Clinical setting

For this study, we have chosen to concentrate on medical devices used in female pelvic floor surgery, to ensure we examine the differences and similarities in ethical and economic considerations that relate to differing classes of device, rather than issues that relate to differences between surgical specialties. Urogynaecology surgery, although a subspecialty of obstetrics and gynaecology, is typical of other surgical specialties which include non-emergency elective surgery.

Pelvic floor disorders (urinary incontinence, fecal incontinence, and pelvic organ prolapse, where pelvic organs bulge into the vagina) are common, affecting at least a third of adult women of all ages [23]. Surgical procedures for pelvic floor disorders are among the most common of all female surgeries [24]. The lifetime risk of undergoing at least one operation for prolapse or incontinence by age 80 is 11% (with 29% of those having at least one re-operation) [25]. The growth in demand for pelvic floor disorder services is expected to increase at twice the rate of population increase, because of the increasing demand for services in women over 60 years of age [26]. Further, the aging "baby boomers" are likely to be more demanding than previous generations [26]. This increasingly important surgical market is seeing intensifying competition between the device manufacturers, leading to a growth in the number of new and minimally invasive surgical devices being developed and marketed.

Expansion in demand for treatments, and concern about the introduction of new technologies, have led to consideration of ethical issues in other surgical areas. For example, there are publications relating to neurosurgery [27], orthopaedics [28], and spine surgery [29], that suggest the issues in other surgical specialties are similar to those experienced in urogynaecology, particularly among surgical procedures that are elective. Similar considerations will apply to non-emergency general surgical procedures such as groin hernia repair and cholecystectomy, although we have not found an examination of the ethical or economic issues associated with the introduction of new devices in these clinical areas.

### Devices

For the purpose of this study, we have chosen three specific devices used in urogynaecology, one for each of the more invasive MDB classes (ie one for each case study) (Table 2). Class I devices will not be included in our study because device manufacturers are not required to obtain individual licences for them, and therefore the licensing requirements and adoption practices are very different.

**Table 2:** Medical devices selected for case study

MDB Class	Device selected for study	Year first licensed
**Class II**	Suture capturing device	- first licensed in 1999
**Class III**	Mesh device for pelvic organ prolapse repair	- first licensed in 2005
**Class IV**	Implantable sacral nerve stimulator	- first licensed in 1999

The choice of these specific devices is based on the premise that slightly older examples are needed for this study, in order that we can examine the types of information that institutions and policy makers used to decide whether to adopt them into practice. Extremely new devices may not have yet found their way into widespread use, and would be less likely to have undergone a thorough evaluation process.

### Stakeholders

Seven groups of stakeholders will be considered for each case study. The groups are those we identified for our initial investigation of the ethical issues associated with the introduction of new surgical devices: regulatory bodies, device manufacturers, clinicians, patients, health care institutions and professional societies [12]. These groups of stakeholders are similar for the adoption of any surgical device into clinical practice, whether urogynaecology, neurosurgery, general surgery or orthopaedics [26-28]. We have also included provincial health departments, because these stakeholders are responsible for health care budgets within provinces, and several provinces have established health technology assessment programs to evaluate new medical devices.

### Principles underlying the study

Ethics and economics are the two disciplines that provide the principles that together underpin this study. The study will be undertaken as an investigation of the conduct and perceptions of the stakeholders using in-depth qualitative interviews. The goal of the research is to provide a rich description of the medical device approval process in the cases considered here, and to begin a public discussion of the strengths and limitations of that process. The interviews will be semi-structured to allow for questions and later analysis related to the ethics and economics principles detailed below. We do not expect each interview respondent or stakeholder group to necessarily or explicitly address these principles. The interview format also allows for respondents to introduce concepts or understandings about the medical device approval process that we have not anticipated. However, we expect at the end of the interview process to be able to discuss the extent to which the following ethical principles and economic principles bear upon the approval and use of the selected medical devices. Given the resource constraints of the health care system, the ethical principles cannot be examined in isolation: both ethics and economics must be addressed together when assessing to what extent reasoned approaches are being taken toward the public good [3]. It is important to identify the relevant ethical and economic principles before starting the research.

### Ethical principles

Four medical ethics principles [3] relevant to the introduction of new surgical devices, will be discussed for each case study, based on our earlier investigation [12]:

**Beneficence**, in the context of this study, is the obligation "*to provide benefit" *to patients [3] in part by ensuring that treatments are effective. **Non-maleficence **is the "*obligation not to inflict harm*" [3] on patients (unless this is outweighed by potential benefit to the patient), in part by providing safe treatments. Beneficence and non-maleficence are inter-related and may be discussed together. In the proposed study, the type and degree of beneficence and non-maleficence vary according to the relationship between specific stakeholders (and their roles) and the patient (or patients). For example, a commonly discussed relationship in medical ethics is that of physician and patient. In this relationship, beneficence is assumed to involve promoting the welfare of specific patients, while non-maleficence is assumed to keep them from harm. In contrast, the discussion of beneficence and non-maleficence as related to the relationship between a health authority and the patients in their region, is also influenced by the need to provide benefit to large numbers of patients with conflicting conditions and needs. The situation relating device manufacturers and patients is clearly even more complex, given that there is not a direct relationship with a specific patient (or specific groups of patients), rather overall obligations of beneficence and non-maleficence directed at patients generally. Further the relationship is conflicted by the obligation to provide profit to the company owners, which influences all aspects of device manufacturers' conduct.

**Respect for autonomy **upholds the patient right of self-determination, the "*right to hold views, to make choices, and to take actions based on personal values and beliefs*" [3]. Such choices include the right to make informed choices about treatment. Doctors also make autonomous decisions, providing the treatment they believe is clinically appropriate, based on patient need, and availability of treatments and resources [3,30]. Thus conflict may occur between respect for the patient's autonomy and the physician's obligation to act in ways he or she believes will be for the patient's medical benefit. Of importance in any discussion about autonomy is the need for patients to be fully informed about the consequences of any treatment choice: in discussions about adoption of new devices into practice, such discussions must include information about lack of evidence, as well as information about outcomes of treatment. As yet, we do not know how much information patients need about the safety and effectiveness of medical devices, how much information physicians give, or whether patients receive the information they need or desire.

**Justice **may be interpreted as "*fair, equitable, and appropriate treatment in light of what is due or owed to persons*" [3]. It is relevant to fair access to treatment, responsible stewardship of scarce resources, and compensation for injury. Conflicts can occur between an individual patient's needs and the needs of larger groups of patients (for example a hospital's patients, or patients in a province) where access to a particular type of treatment may be restricted as a result of limited resources.

Thus the ethical perspective within the context of health care provision changes depending on specific relationships between stakeholders and patients. However, these ethical relationships are not unique to urogynaecology: rather the situations observed in urogynaecology are representative of other surgical specialties and subspecialties.

### Economic principles

The main economic principles to be examined are those concerned with the introduction of new technologies into a healthcare market [31].

### Scarcity of resources

Resources are limited in the health care system, both within urogynaecology itself, and more widely within institutions and provincial health care [31]. Allocation of scarce resources within drug treatment budgets is well defined: drugs may be paid for within provincial health care systems only if they are included within provincial drug benefit programs [32,33]. Payment for surgical procedures is also determined provincially, but the situation for specific surgical devices depends on local decisions by individual institutions, at hospital or regional health authority level. Deliberations about allocation of scarce resources may be enhanced by the availability of relevant health technology assessments.

### Choice

In situations involving limited resources, demand may outstrip supply, therefore choices need to be made to allocate them [34]. Choices relating to specific surgical devices are made locally, by hospitals or regional health authorities, based on evidence from manufacturers, clinicians and health technology evaluations (if available). Even if a new device has clear evidence of benefit, it is possible that a hospital will choose not to adopt it because of resource constraints. Budgetary choices will be made in the context of hospital, health authority or provincial resources, and choices between clinical areas (and type of patient) may be extremely difficult. Such decisions may potentially lead to equity issues if resources are available only in selected institutions or to specific patients.

### Opportunity costs

These describe the cost of actions that lead to opportunities forgone [34,35]. Local choices regarding specific medical devices may depend on the need to divert resources from one budgetary area to another. In the case of urogynaecology, choices may impact on other surgical procedures within gynaecology, may impact on other surgical specialties, or more generally on the health budget. The evaluation of opportunity costs in the context of limited resources may have significant impact on choice in health care settings [36].

In the setting of urogynaecology device licensing, marketing and use, discussion of these economic principles will contrast widely between the individual stakeholders. Economic principles cannot be discussed in isolation: the disciplines of ethics and economics are fundamental to the allocation of scarce resources and fairness to patients [3,5], alongside the assessment of clinical effectiveness.

### Interviews with stakeholders

Stakeholder interviews will be approached using an in-depth, semi-structured approach. The interviews will have some prepared, open-ended questions (with probes and follow-ups also prepared to focus the interview when needed), but respondents will also be able to introduce issues and concerns not included in the interview guide. Therefore, although the researchers are entering the interview conversation with a partial conceptual framework based upon the ethical and economic principles defined above, the interview guides as well as final analysis will evolve from the collection and interpretation of the data. In addition, the interviews will focus on two inter-related but analytically distinct dimensions: the respondents' *descriptions *of the current process through which the selected medical devices are approved and put into practice (for each class of device, as relevant to respondent), and their *feelings *or *perceptions *about that process and how it might be improved. For instance, what are the roles and responsibilities of various stakeholders in the process? What kind of information do they have at hand for decision-making and what kind of information or policy do they think they need? Stakeholder-specific interview schedules will be developed: interviews will be in-depth guided by the interview schedule.

Despite the in-depth nature of the interviews, this type of interviewing should still be called "investigative interviewing" because the topic has a fairly narrow scope (how new medical devices come into practice) and focuses on events and processes (rather than, for instance, cultural meanings, life histories, or theory elaboration) [37]. In-depth interviews are a suitable approach for this study, since the stakeholders inhabit different work and life settings with very different perspectives and priorities in relation to licensing and adopting new medical devices. Such perspectives are difficult to capture using standardized survey or interview instrument.

Interviews will be conducted face-to-face in the office of the interviewee. They will be scheduled to take an hour, and be audio recorded and transcribed, with field notes taken by the interviewer. Interviews will be conducted for each of the stakeholder groups listed overleaf. The researchers will stop interviewing when either there are no appropriate respondents remaining in the stakeholder group (which will happen when the group is quite small), or when respondents within each group begin to substantially duplicate the answers other respondents have already given. The latter is known as the "saturation point."

In terms of coding and analysis of the interview data, the goal of the research is to examine the responses from all the stakeholder groups and identify similarities and differences within and between their accounts [38,39]. The researchers will be able to report their ideas about problems with the current process and potential solutions, but will also be in a position to form initial, normative interpretations of potential weaknesses in the system based on an analysis of all the interviews. Juxtaposing the interviews from the different stakeholder groups also enables the researchers to identify and perhaps explain differing perceptions of the actual process by which medical devices are licensed, regulated and put into practice in Canada, as well as potential obstacles to improving that process.

The following individual stakeholders will be approached for interview.

### Representatives from Health Canada's MDB

Representatives of the MDB, the Device Licensing Services Division, and Marketed Health Products Directorate will be invited to comment on the current and evolving position of Health Canada in relation to the regulation of new devices.

### Representatives of device manufacturers

Product development and marketing leaders from the relevant device manufacturers, will be interviewed about the roles, responsibilities, and information and policy needs believed to be important when introducing new medical devices.

### Clinician representatives

Clinicians from urogynaecology divisions in two Canadian provinces will be asked to describe the system for adopting new technologies in their institution, and their own roles and responsibilities in adopting new devices.

### Patient representatives

Patients recruited from pelvic floor clinics in two Canadian provinces, will be interviewed to determine the information patients expect would be available for new medical devices used in urogynaecology, and who should be providing and evaluating the information.

### Health care institution representatives

Individuals responsible for overseeing the introduction of new surgical technologies (for example, executive chairs of new technology committees, heads of Departments of Obstetrics and Gynaecology and Surgery) in two Canadian provinces, will be interviewed about the roles, responsibilities, and information and policy needs believed by the interviewees to be important when introducing new medical devices.

### Provincial health departments

Representatives of the two Canadian provincial health departments who are responsible for consultation on new medical devices will be interviewed, about the roles, responsibilities, and information and policy needs believed to be important when introducing new medical devices.

### Professional societies

Key individuals in professional societies and associations representing the Canadian medical device industry will be interviewed about the perceived role of these societies in the introduction of new medical devices.

### Data analysis

Data analysis will be undertaken for each group of stakeholders, taking the perspective of that stakeholder. Analyses will be conducted by first reading and re-reading the transcribed interviews, seeking to understand stakeholders' views of the roles, responsibilities, and information and policy needs believed by the interviewees to be important when introducing new medical devices. Views of their own and other stakeholders' roles will be important to incorporate. After achieving familiarity with the data, analysis codes will be assigned to the transcribed text, and analysis will seek to identify themes that are relevant to the study [28]. An analysis will be conducted for each class of device and each type of stakeholder.

### Synthesis and interpretation of information from the case studies

One final report will be prepared for each class of device, with stakeholder issues discussed for each device. For each class of device, a descriptive subsection will examine the stakeholders' roles, responsibilities, and information and policy needs in adopting new devices into clinical practice. Applied ethics theory, analysis and critical interpretation will be used to further illuminate the case study material.

Stakeholders are likely to have contrasting and competing ethical and economic perspectives, particularly with regard to the higher risk categories of surgical devices. The goal of the study is not to reconcile these approaches, but rather to understand how they interact, and how they influence the activities of the stakeholders.

Final reports for each device class will include descriptions of the ethical and economic considerations that impact on the regulation and adoption of such devices into clinical practice, and will, if possible, also make recommendations for future conduct of stakeholders.

## Discussion

The main value of this research will be to describe the current situation as it relates to the adoption of new surgical devices into clinical practice in Canada. It appears from our initial analysis that, in a rapidly developing area of surgical treatment, the regulation and adoption of new devices is only partially guided by ethical considerations [12]. Further, different stakeholders have different perspectives and goals. Thus a comprehensive investigation is required to identify the important ethical and economic issues that guide the behaviours of stakeholders including manufacturers, regulators, clinicians and patients. The proposed research will provide that comprehensive analysis.

Although the research will be undertaken in Canada, given the similarity between the regulatory requirements in the USA and European community, the findings of the study will be relevant to other jurisdictions.

The research will highlight possible deficiencies in the current system of regulation and adoption of new medical devices. For example, it is possible that the research will find that MDB does not widely communicate its role in the regulation of medical devices, and that therefore MDB is perceived by stakeholders as being more "responsible" for the safety of therapeutic medical devices than is currently possible based on economic constraints. If the research does identify such a problem, improvement in communication about MDB's role may increase the level of understanding by clinicians and patients. Another example might be that physician communication with patients about the level of evidence and experience with new devices is lacking. If this proves to be the case, then further research will be undertaken to determine what type of communication is best under such circumstances.

The research will provide evidence by which to guide future behaviour of stakeholders, either by changing the focus of behaviour in adopting new surgical devices (for example in seeking robust evidence before adopting a new medical device), or else by changing behaviours to communicate and highlight the true availability of evidence of safety and effectiveness. The final stage of the research will consist of a dissemination phase, during which the research team will communicate the findings of the research widely to stakeholders.

Thus we believe the value of the research findings can be leveraged by knowledge translation into improved practice (if necessary) by the stakeholders, as well as further research in an underdeveloped research area.

## Competing interests

The authors declare that they have no competing interests.

## Authors' contributions

SR played a major role in the conception and design of the study. She will lead the study, will be involved in interviewing participants, and will undertake analysis and interpretation of data. CW played a major role in the conception and design of the study. He will lead the ethics aspects of the study. AG played a major role in the conception and design of the study. He will lead the economic aspects of the study. AD played a major role in the design of the study. She will provide the qualitative and ethnographic expertise. She will be involved in the collection, analysis, and interpretation of data. CT played a major role in the design of the study. She will be involved in the collection, analysis, and interpretation of data. RL played a major role in the design of the study. His expertise spans clinical, policy making and knowledge transfer interests, and he will lead the study in these areas. All authors have read and given final approval of the manuscript as submitted.

## Authors' Information

SR is a health services researcher and Director of Research in the University of Calgary Department of Obstetrics and Gynaecology. CW is a research ethicist and Canada Research Chair in the Department of Philosophy at the University of Western Ontario, and Director of the Joseph L Rotman Institute of Science and Values. AG is a health economist and Professor in the Department of Clinical Epidemiology and Biostatistics at McMaster University. AD is a sociologist and Assistant Professor in the University of Calgary Department of Sociology, and specializes in the social science of medical knowledge and technologies, health care policy and political economy. CT is a graduate student in Applied Psychology at the University of Calgary. RL is a general surgeon, and Professor in the Department of Surgery at the University of Calgary. He is a past President of the Canadian Association of General Surgeons (CAGS).

## Pre-publication history

The pre-publication history for this paper can be accessed here:

http://www.biomedcentral.com/1472-6939/11/14/prepub
